# Bis[1,3-bis­(benzimidazol-2-yl)-2-oxa­propane]cobalt(II) dipicrate acetonitrile tris­olvate

**DOI:** 10.1107/S1600536809024234

**Published:** 2009-07-01

**Authors:** Huilu Wu, Ruirui Yun, Xingcai Huang, Qingyu Sun, Baoliang Qi

**Affiliations:** a School of Chemical and Biological Engineering, Lanzhou Jiaotong University, Lanzhou 730070, People’s Republic of China; b School of Chemical and Biological Engineering, Lanzhou Jiaotong University, Lanzhou 730070, People’s Republic of China

## Abstract

In the title compound, [Co(C_16_H_14_N_4_O)_2_](C_6_H_2_N_3_O_7_)_2_·3CH_3_CN, the Co^II^ ion is located on a crystallographic twofold rotation axis and is coordinated in a slightly distorted tetra­hedral environment by four N atoms from the two bidentate *N*-heterocycles. The crystal structure is stabilized by inter­molecular N—H⋯O and N—H⋯N hydrogen bonds. One of the acetonitrile solvent mol­ecules also lies on a twofold rotation axis.

## Related literature

For the crystal structures of related dipicrate metal complexes with 1,3-bis­(1-benzyl-1H-benzimidazol-2-yl)-2-oxapropane ligands, see: Wu, Yun, Li, Wang & Huang (2009 ▶); Wu, Yun, Li, Tao & Wang (2009 ▶).
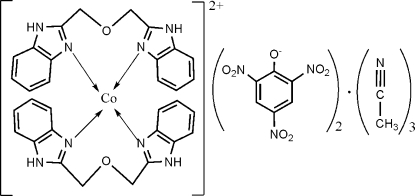


## Experimental

### 

#### Crystal data

[Co(C_16_H_14_N_4_O)_2_](C_6_H_2_N_3_O_7_)_2_·3C_2_H_3_N
*M* *_r_* = 1194.93
Monoclinic, 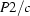

*a* = 11.4114 (3) Å
*b* = 9.9303 (2) Å
*c* = 25.1442 (6) Å
β = 111.164 (1)°
*V* = 2657.12 (11) Å^3^
*Z* = 2
Mo *K*α radiation
μ = 0.41 mm^−1^
*T* = 153 K
0.28 × 0.25 × 0.17 mm

#### Data collection

Rigaku R-AXIS Spider diffractometer
Absorption correction: multi-scan (*ABSCOR*; Higashi, 1995 ▶) *T*_min_ = 0.894, *T*_max_ = 0.933
25278 measured reflections
6093 independent reflections
5418 reflections with *I* > 2σ(*I*)
*R*_int_ = 0.021

#### Refinement

*R*[*F* ^2^ > 2σ(*F* ^2^)] = 0.031
*wR*(*F* ^2^) = 0.087
*S* = 1.04
6093 reflections
392 parameters
2 restraints
H atoms treated by a mixture of independent and constrained refinement
Δρ_max_ = 0.45 e Å^−3^
Δρ_min_ = −0.30 e Å^−3^

### 

Data collection: *RAPID-AUTO* (Rigaku/MSC, 2004 ▶); cell refinement: *RAPID-AUTO*; data reduction: *RAPID-AUTO*; program(s) used to solve structure: *SHELXS97* (Sheldrick, 2008 ▶); program(s) used to refine structure: *SHELXL97* (Sheldrick, 2008 ▶); molecular graphics: *SHELXTL* (Sheldrick, 2008 ▶); software used to prepare material for publication: *SHELXTL*.

## Supplementary Material

Crystal structure: contains datablocks global, I. DOI: 10.1107/S1600536809024234/lh2846sup1.cif

Structure factors: contains datablocks I. DOI: 10.1107/S1600536809024234/lh2846Isup2.hkl

Additional supplementary materials:  crystallographic information; 3D view; checkCIF report

## Figures and Tables

**Table 1:** Hydrogen-bond geometry (Å, °)

*D*—H⋯*A*	*D*—H	H⋯*A*	*D*⋯*A*	*D*—H⋯*A*
N2—H2*N*⋯O6^i^	0.858 (9)	1.893 (12)	2.6746 (14)	150.7 (17)
N2—H2*N*⋯O5^i^	0.858 (9)	2.381 (15)	3.0166 (15)	131.3 (15)
N4—H4*N*⋯N8	0.858 (9)	2.052 (10)	2.9040 (18)	171.8 (18)

Symmetry code: (i) 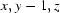.

**Fig. 1.**
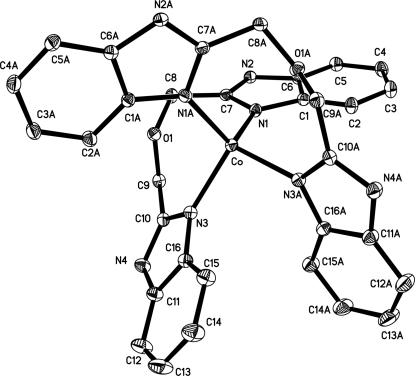
Molecular structure and atom numbering for the cation of the title compound. Hydrogen atoms have been omitted for clarity and the displacement ellipsoids are shown at the 30% probability level [symmetry code: (A) -x+1, y, -z+3/2].

**Fig. 2.**
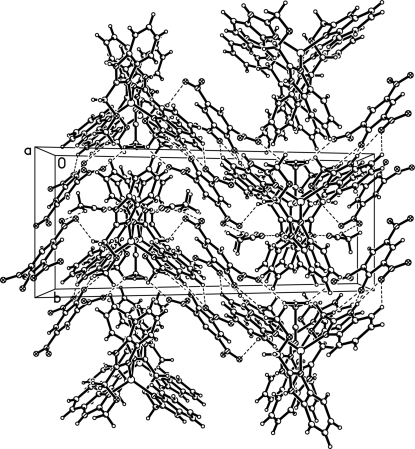
Part of the crystal structure with hydrogen bonds shown as dashed lines.

## supplementary crystallographic information

### Comment

The asymmetric unit of the title compound consists of half a discrete di[1,3-bis(benzimidazol-2-yl)-2-oxopropane] cobalt(II) cation, one picrate anion and 1.5 molecules of acetonitrile; the formula unit is generated by a twofold rotation axis. The cation is shown in Fig. 1. The Co^II^ ion is four-coordinate with a N_4_ ligand set. The (1,3-bis(benzimidazol-2-yl)-2-oxopropane) ligand acts as a bidentate donor. The coordination geometry of the Co^II^ may be best described as slightly distorted tetrahedral. This geometry is assumed by the Co^II^ to relieve the steric crowding. The crystal structure is stabilized by intermolecular N—H···O and N—H···N hydrogen bonds. Additional stabilization is provided by weak intermolecular C-H···O hydrogen bonds (Fig. 2).

### Experimental

To a stirred solution of 1,3-bis(benzimidazol-2-yl)-2-oxopropane (0.139 g, 0.5 mmol) in hot MeOH (15 ml) was added Co(C_6_H_2_N_3_O_7_)_2_ (0.129 g, 0.25 mmol) in MeOH (5 ml). A red crystalline product formed rapidly. The precipitate was filtered off, washed with MeOH and absolute Et_2_O, and dried *in vacuo*. The dried precipitate was dissolved in acetonitrile to form a red solution that was allowed to evaporate at room temperature. Red crystals suitable for X-ray diffraction studies were obtained after three days at room temperature. Yield, 0.106 g (66%). (found: C, 50.20; H, 3.51; N,19.94. Calcd. for C50H41N17O16Co: C, 50.26; H, 3.46; N, 19.93)

### Refinement

All H atoms were found in difference electron maps and were subsequently included in a riding-model approximation with C—H distances ranging from 0.95 to 0.99 Å and *U*_iso_(H) = 1.2 *U*_eq_ of the carrier atom. The H atoms bonded to N atoms were refined independently with the distance constraint of N—H 0.858 (9) Å.

### Crystal data

**Table:**

[Co(C_16_H_14_N_4_O)_2_](C_6_H_2_N_3_O_7_)_2_·3C_2_H_3_N	*F*(000) = 1230
*M**_r_* = 1194.93	*D*_x_ = 1.494 Mg m^−^^3^
Monoclinic, *P*2/*c*	Mo *K*α radiation, λ = 0.71073 Å
Hall symbol: -P 2yc	Cell parameters from 7749 reflections
*a* = 11.4114 (3) Å	θ = 3.2–27.5°
*b* = 9.9303 (2) Å	µ = 0.41 mm^−^^1^
*c* = 25.1442 (6) Å	*T* = 153 K
β = 111.164 (1)°	Block, red
*V* = 2657.12 (11) Å^3^	0.28 × 0.25 × 0.17 mm
*Z* = 2	

### Data collection

**Table:**

Rigaku R-AXIS Spider diffractometer	6093 independent reflections
Radiation source: fine-focus sealed tube	5418 reflections with *I* > 2σ(*I*)
graphite	*R*_int_ = 0.021
φ and ω scans	θ_max_ = 27.5°, θ_min_ = 3.0°
Absorption correction: multi-scan (*ABSCOR*; Higashi, 1995)	*h* = −14→14
*T*_min_ = 0.894, *T*_max_ = 0.933	*k* = −12→12
25278 measured reflections	*l* = −32→32

### Refinement

**Table:**

Refinement on *F*^2^	Secondary atom site location: difference Fourier map
Least-squares matrix: full	Hydrogen site location: inferred from neighbouring sites
*R*[*F*^2^ > 2σ(*F*^2^)] = 0.031	H atoms treated by a mixture of independent and constrained refinement
*wR*(*F*^2^) = 0.087	*w* = 1/[σ^2^(*F*_o_^2^) + (0.0487*P*)^2^ + 0.9118*P*] where *P* = (*F*_o_^2^ + 2*F*_c_^2^)/3
*S* = 1.03	(Δ/σ)_max_ = 0.001
6093 reflections	Δρ_max_ = 0.45 e Å^−^^3^
392 parameters	Δρ_min_ = −0.30 e Å^−^^3^
2 restraints	Extinction correction: *SHELXL97* (Sheldrick, 2008), Fc^*^=kFc[1+0.001xFc^2^λ^3^/sin(2θ)]^-1/4^
Primary atom site location: structure-invariant direct methods	Extinction coefficient: 0.0036 (5)

### Special details

**Table:**

Geometry. All e.s.d.'s (except the e.s.d. in the dihedral angle between two l.s. planes) are estimated using the full covariance matrix. The cell e.s.d.'s are taken into account individually in the estimation of e.s.d.'s in distances, angles and torsion angles; correlations between e.s.d.'s in cell parameters are only used when they are defined by crystal symmetry. An approximate (isotropic) treatment of cell e.s.d.'s is used for estimating e.s.d.'s involving l.s. planes.
Refinement. Refinement of *F*^2^ against ALL reflections. The weighted *R*-factor *wR* and goodness of fit *S* are based on *F*^2^, conventional *R*-factors *R* are based on *F*, with *F* set to zero for negative *F*^2^. The threshold expression of *F*^2^ > σ(*F*^2^) is used only for calculating *R*-factors(gt) *etc*. and is not relevant to the choice of reflections for refinement. *R*-factors based on *F*^2^ are statistically about twice as large as those based on *F*, and *R*- factors based on ALL data will be even larger.

### Fractional atomic coordinates and isotropic or equivalent isotropic displacement parameters (Å^2^)

**Table:**

	*x*	*y*	*z*	*U*_iso_*/*U*_eq_	Occ. (<1)
Co	0.5000	0.36301 (2)	0.7500	0.01700 (8)	
O1	0.67337 (8)	0.24981 (9)	0.71220 (4)	0.02259 (19)	
O2	0.74591 (13)	0.49084 (15)	0.46620 (6)	0.0583 (4)	
O3	0.54824 (12)	0.47322 (11)	0.41556 (5)	0.0409 (3)	
O4	0.25535 (11)	0.76959 (16)	0.44436 (6)	0.0567 (4)	
O5	0.29928 (11)	0.88294 (12)	0.52194 (5)	0.0428 (3)	
O6	0.54003 (9)	0.96666 (10)	0.56756 (4)	0.0292 (2)	
O7	0.78254 (12)	0.91568 (16)	0.63960 (5)	0.0522 (3)	
O8	0.88585 (11)	0.89860 (13)	0.58266 (5)	0.0420 (3)	
N1	0.41234 (10)	0.25020 (10)	0.67914 (4)	0.0200 (2)	
N2	0.39034 (10)	0.10735 (11)	0.60795 (4)	0.0202 (2)	
N3	0.62918 (10)	0.49981 (11)	0.74562 (4)	0.0195 (2)	
N4	0.77113 (11)	0.58828 (12)	0.71442 (5)	0.0260 (2)	
N5	0.63674 (13)	0.52737 (13)	0.45326 (5)	0.0342 (3)	
N6	0.33090 (11)	0.81256 (13)	0.48951 (5)	0.0309 (3)	
N7	0.79113 (12)	0.88240 (13)	0.59422 (5)	0.0332 (3)	
N8	0.93174 (15)	0.59702 (16)	0.64743 (6)	0.0452 (4)	
N9	1.0000	0.3626 (2)	0.7500	0.0471 (5)	
C1	0.28403 (12)	0.23999 (12)	0.64561 (5)	0.0196 (2)	
C2	0.17845 (12)	0.29913 (14)	0.65151 (5)	0.0252 (3)	
H2	0.1860	0.3604	0.6816	0.030*	
C3	0.06220 (13)	0.26482 (15)	0.61173 (6)	0.0282 (3)	
H3	−0.0113	0.3029	0.6150	0.034*	
C4	0.04975 (13)	0.17528 (15)	0.56674 (6)	0.0278 (3)	
H4	−0.0317	0.1546	0.5402	0.033*	
C5	0.15352 (13)	0.11673 (14)	0.56030 (6)	0.0249 (3)	
H5	0.1458	0.0568	0.5298	0.030*	
C6	0.26998 (12)	0.14996 (12)	0.60080 (5)	0.0194 (2)	
C7	0.47004 (12)	0.16761 (12)	0.65481 (5)	0.0197 (2)	
C8	0.60830 (12)	0.13872 (13)	0.67854 (6)	0.0252 (3)	
H8A	0.6244	0.0564	0.7023	0.030*	
H8B	0.6391	0.1231	0.6469	0.030*	
C9	0.68619 (12)	0.35957 (13)	0.67806 (5)	0.0222 (3)	
H9A	0.6121	0.3650	0.6422	0.027*	
H9B	0.7624	0.3481	0.6684	0.027*	
C10	0.69630 (11)	0.48306 (13)	0.71274 (5)	0.0201 (2)	
C11	0.75372 (13)	0.68185 (14)	0.75169 (6)	0.0267 (3)	
C12	0.80865 (17)	0.80687 (16)	0.77019 (7)	0.0396 (4)	
H12	0.8685	0.8446	0.7562	0.047*	
C13	0.77203 (19)	0.87304 (16)	0.80967 (8)	0.0450 (4)	
H13	0.8071	0.9588	0.8232	0.054*	
C14	0.68394 (17)	0.81668 (16)	0.83043 (7)	0.0393 (4)	
H14	0.6612	0.8653	0.8578	0.047*	
C15	0.62941 (14)	0.69269 (15)	0.81217 (6)	0.0293 (3)	
H15	0.5702	0.6549	0.8265	0.035*	
C16	0.66544 (13)	0.62542 (13)	0.77169 (5)	0.0221 (3)	
C17	0.61070 (14)	0.63884 (13)	0.48431 (6)	0.0262 (3)	
C18	0.48772 (13)	0.67268 (13)	0.47497 (5)	0.0238 (3)	
H18	0.4208	0.6222	0.4489	0.029*	
C19	0.46257 (13)	0.78010 (13)	0.50371 (5)	0.0233 (3)	
C20	0.55850 (13)	0.86220 (13)	0.54417 (5)	0.0228 (3)	
C21	0.68386 (13)	0.81456 (14)	0.55233 (6)	0.0260 (3)	
C22	0.70968 (14)	0.71058 (15)	0.52261 (6)	0.0288 (3)	
H22	0.7941	0.6878	0.5281	0.035*	
C23	1.05744 (17)	0.6226 (2)	0.58074 (8)	0.0495 (5)	
H23A	1.0127	0.5755	0.5449	0.059*	
H23B	1.1415	0.5837	0.5987	0.059*	
H23C	1.0648	0.7183	0.5730	0.059*	
C24	0.98832 (15)	0.60810 (18)	0.61876 (7)	0.0371 (4)	
C25	1.0000	0.1051 (3)	0.7500	0.0971 (16)	
H25A	1.0691	0.0722	0.7836	0.116*	0.50
H25B	1.0110	0.0722	0.7154	0.116*	0.50
H25C	0.9199	0.0722	0.7511	0.116*	0.50
C26	1.0000	0.2497 (2)	0.7500	0.0355 (5)	
H2N	0.4130 (16)	0.0525 (15)	0.5871 (7)	0.037 (5)*	
H4N	0.8242 (14)	0.5947 (19)	0.6975 (7)	0.041 (5)*	

### Atomic displacement parameters (Å^2^)

**Table:**

	*U*^11^	*U*^22^	*U*^33^	*U*^12^	*U*^13^	*U*^23^
Co	0.01758 (12)	0.01751 (13)	0.01522 (12)	0.000	0.00511 (9)	0.000
O1	0.0227 (5)	0.0215 (4)	0.0208 (4)	−0.0016 (3)	0.0046 (4)	−0.0025 (3)
O2	0.0533 (8)	0.0616 (9)	0.0637 (8)	0.0260 (7)	0.0257 (7)	−0.0152 (7)
O3	0.0612 (8)	0.0328 (6)	0.0310 (5)	0.0086 (5)	0.0194 (5)	−0.0069 (5)
O4	0.0274 (6)	0.0785 (10)	0.0554 (8)	−0.0016 (6)	0.0044 (6)	−0.0380 (7)
O5	0.0291 (6)	0.0489 (7)	0.0506 (7)	0.0037 (5)	0.0147 (5)	−0.0249 (6)
O6	0.0275 (5)	0.0275 (5)	0.0321 (5)	0.0021 (4)	0.0102 (4)	−0.0101 (4)
O7	0.0354 (6)	0.0790 (10)	0.0353 (6)	0.0055 (6)	0.0046 (5)	−0.0223 (6)
O8	0.0292 (6)	0.0469 (7)	0.0486 (7)	−0.0026 (5)	0.0126 (5)	0.0015 (5)
N1	0.0186 (5)	0.0204 (5)	0.0200 (5)	0.0016 (4)	0.0058 (4)	−0.0023 (4)
N2	0.0214 (5)	0.0189 (5)	0.0203 (5)	0.0001 (4)	0.0075 (4)	−0.0037 (4)
N3	0.0211 (5)	0.0207 (5)	0.0178 (5)	−0.0001 (4)	0.0082 (4)	−0.0005 (4)
N4	0.0287 (6)	0.0261 (6)	0.0286 (6)	−0.0045 (5)	0.0170 (5)	−0.0010 (5)
N5	0.0495 (8)	0.0292 (6)	0.0304 (6)	0.0138 (6)	0.0222 (6)	0.0023 (5)
N6	0.0251 (6)	0.0324 (6)	0.0345 (6)	0.0000 (5)	0.0100 (5)	−0.0109 (5)
N7	0.0271 (6)	0.0361 (7)	0.0327 (6)	0.0065 (5)	0.0061 (5)	−0.0024 (5)
N8	0.0477 (9)	0.0524 (9)	0.0459 (8)	0.0125 (7)	0.0295 (7)	0.0123 (7)
N9	0.0421 (12)	0.0387 (12)	0.0568 (13)	0.000	0.0132 (10)	0.000
C1	0.0192 (6)	0.0204 (6)	0.0178 (5)	−0.0006 (5)	0.0052 (5)	−0.0001 (4)
C2	0.0217 (6)	0.0300 (7)	0.0234 (6)	0.0023 (5)	0.0074 (5)	−0.0052 (5)
C3	0.0199 (6)	0.0351 (7)	0.0289 (7)	0.0021 (5)	0.0080 (5)	−0.0046 (6)
C4	0.0203 (6)	0.0330 (7)	0.0259 (6)	−0.0026 (5)	0.0035 (5)	−0.0040 (6)
C5	0.0247 (7)	0.0264 (6)	0.0215 (6)	−0.0022 (5)	0.0059 (5)	−0.0051 (5)
C6	0.0204 (6)	0.0191 (6)	0.0196 (5)	−0.0003 (4)	0.0082 (5)	0.0008 (4)
C7	0.0212 (6)	0.0167 (5)	0.0209 (6)	−0.0001 (5)	0.0073 (5)	−0.0009 (4)
C8	0.0206 (6)	0.0220 (6)	0.0294 (6)	0.0019 (5)	0.0049 (5)	−0.0078 (5)
C9	0.0217 (6)	0.0264 (7)	0.0198 (6)	0.0004 (5)	0.0091 (5)	−0.0015 (5)
C10	0.0189 (6)	0.0239 (6)	0.0179 (5)	0.0011 (5)	0.0073 (5)	0.0026 (5)
C11	0.0299 (7)	0.0246 (7)	0.0280 (6)	−0.0030 (5)	0.0133 (6)	−0.0004 (5)
C12	0.0469 (9)	0.0293 (8)	0.0490 (9)	−0.0132 (7)	0.0251 (8)	−0.0049 (7)
C13	0.0582 (11)	0.0270 (8)	0.0540 (10)	−0.0149 (7)	0.0254 (9)	−0.0139 (7)
C14	0.0509 (10)	0.0305 (8)	0.0415 (8)	−0.0047 (7)	0.0228 (8)	−0.0134 (7)
C15	0.0347 (8)	0.0284 (7)	0.0283 (6)	−0.0023 (6)	0.0156 (6)	−0.0056 (6)
C16	0.0240 (6)	0.0213 (6)	0.0211 (6)	−0.0010 (5)	0.0080 (5)	−0.0001 (5)
C17	0.0380 (8)	0.0225 (6)	0.0229 (6)	0.0085 (5)	0.0168 (6)	0.0030 (5)
C18	0.0335 (7)	0.0202 (6)	0.0203 (6)	0.0012 (5)	0.0130 (5)	0.0008 (5)
C19	0.0259 (7)	0.0229 (6)	0.0229 (6)	0.0037 (5)	0.0111 (5)	−0.0006 (5)
C20	0.0256 (7)	0.0230 (6)	0.0216 (6)	0.0032 (5)	0.0106 (5)	0.0002 (5)
C21	0.0252 (7)	0.0282 (7)	0.0238 (6)	0.0038 (5)	0.0081 (5)	0.0003 (5)
C22	0.0287 (7)	0.0332 (7)	0.0269 (6)	0.0109 (6)	0.0129 (6)	0.0036 (6)
C23	0.0350 (9)	0.0802 (14)	0.0412 (9)	0.0146 (9)	0.0233 (8)	0.0109 (9)
C24	0.0319 (8)	0.0471 (9)	0.0344 (8)	0.0142 (7)	0.0146 (7)	0.0100 (7)
C25	0.109 (3)	0.0309 (15)	0.110 (3)	0.000	−0.011 (3)	0.000
C26	0.0263 (10)	0.0338 (12)	0.0407 (11)	0.000	0.0052 (9)	0.000

### Geometric parameters (Å, °)

**Table:**

Co—N1	2.0355 (10)	C4—H4	0.9500
Co—N1^i^	2.0355 (10)	C5—C6	1.3910 (18)
Co—N3	2.0367 (11)	C5—H5	0.9500
Co—N3^i^	2.0367 (11)	C7—C8	1.4993 (18)
O1—C8	1.4250 (15)	C8—H8A	0.9900
O1—C9	1.4272 (16)	C8—H8B	0.9900
O2—N5	1.2234 (19)	C9—C10	1.4849 (18)
O3—N5	1.2315 (18)	C9—H9A	0.9900
O4—N6	1.2278 (17)	C9—H9B	0.9900
O5—N6	1.2233 (16)	C11—C12	1.393 (2)
O6—C20	1.2475 (16)	C11—C16	1.3956 (19)
O7—N7	1.2255 (17)	C12—C13	1.375 (2)
O8—N7	1.2270 (17)	C12—H12	0.9500
N1—C7	1.3308 (16)	C13—C14	1.405 (3)
N1—C1	1.4048 (16)	C13—H13	0.9500
N2—C7	1.3416 (16)	C14—C15	1.381 (2)
N2—C6	1.3855 (16)	C14—H14	0.9500
N2—H2N	0.858 (9)	C15—C16	1.3977 (19)
N3—C10	1.3247 (15)	C15—H15	0.9500
N3—C16	1.4004 (16)	C17—C18	1.378 (2)
N4—C10	1.3404 (17)	C17—C22	1.388 (2)
N4—C11	1.3840 (18)	C18—C19	1.3756 (18)
N4—H4N	0.858 (9)	C18—H18	0.9500
N5—C17	1.4459 (17)	C19—C20	1.4469 (19)
N6—C19	1.4497 (18)	C20—C21	1.4492 (19)
N7—C21	1.4597 (19)	C21—C22	1.368 (2)
N8—C24	1.133 (2)	C22—H22	0.9500
N9—C26	1.121 (3)	C23—C24	1.450 (2)
C1—C2	1.3956 (18)	C23—H23A	0.9800
C1—C6	1.4013 (17)	C23—H23B	0.9800
C2—C3	1.3850 (19)	C23—H23C	0.9800
C2—H2	0.9500	C25—C26	1.436 (4)
C3—C4	1.405 (2)	C25—H25A	0.9800
C3—H3	0.9500	C25—H25B	0.9800
C4—C5	1.380 (2)	C25—H25C	0.9800
			
N1—Co—N1^i^	113.22 (6)	C10—C9—H9A	110.5
N1—Co—N3	117.15 (4)	O1—C9—H9B	110.5
N1^i^—Co—N3	106.14 (4)	C10—C9—H9B	110.5
N1—Co—N3^i^	106.13 (4)	H9A—C9—H9B	108.7
N1^i^—Co—N3^i^	117.15 (4)	N3—C10—N4	112.81 (11)
N3—Co—N3^i^	96.33 (6)	N3—C10—C9	122.03 (11)
C8—O1—C9	112.25 (10)	N4—C10—C9	125.15 (11)
C7—N1—C1	105.13 (10)	N4—C11—C12	131.85 (13)
C7—N1—Co	124.97 (9)	N4—C11—C16	105.85 (12)
C1—N1—Co	129.90 (8)	C12—C11—C16	122.27 (13)
C7—N2—C6	107.67 (10)	C13—C12—C11	116.71 (15)
C7—N2—H2N	124.2 (12)	C13—C12—H12	121.6
C6—N2—H2N	128.2 (12)	C11—C12—H12	121.6
C10—N3—C16	105.33 (10)	C12—C13—C14	121.49 (14)
C10—N3—Co	122.88 (9)	C12—C13—H13	119.3
C16—N3—Co	131.77 (8)	C14—C13—H13	119.3
C10—N4—C11	107.41 (11)	C15—C14—C13	121.90 (14)
C10—N4—H4N	126.7 (13)	C15—C14—H14	119.0
C11—N4—H4N	125.8 (13)	C13—C14—H14	119.0
O2—N5—O3	123.27 (13)	C14—C15—C16	116.89 (14)
O2—N5—C17	118.13 (14)	C14—C15—H15	121.6
O3—N5—C17	118.60 (13)	C16—C15—H15	121.6
O5—N6—O4	122.50 (13)	C11—C16—C15	120.73 (13)
O5—N6—C19	119.46 (12)	C11—C16—N3	108.58 (11)
O4—N6—C19	118.03 (12)	C15—C16—N3	130.67 (12)
O7—N7—O8	123.97 (14)	C18—C17—C22	121.11 (12)
O7—N7—C21	117.91 (13)	C18—C17—N5	119.33 (13)
O8—N7—C21	118.07 (12)	C22—C17—N5	119.56 (13)
C2—C1—C6	120.09 (12)	C19—C18—C17	119.49 (13)
C2—C1—N1	131.19 (11)	C19—C18—H18	120.3
C6—C1—N1	108.69 (11)	C17—C18—H18	120.3
C3—C2—C1	117.27 (12)	C18—C19—C20	123.90 (12)
C3—C2—H2	121.4	C18—C19—N6	115.93 (12)
C1—C2—H2	121.4	C20—C19—N6	120.12 (11)
C2—C3—C4	121.95 (13)	O6—C20—C19	125.89 (12)
C2—C3—H3	119.0	O6—C20—C21	122.06 (13)
C4—C3—H3	119.0	C19—C20—C21	111.92 (11)
C5—C4—C3	121.29 (12)	C22—C21—C20	124.43 (13)
C5—C4—H4	119.4	C22—C21—N7	116.92 (13)
C3—C4—H4	119.4	C20—C21—N7	118.64 (12)
C4—C5—C6	116.58 (12)	C21—C22—C17	119.00 (13)
C4—C5—H5	121.7	C21—C22—H22	120.5
C6—C5—H5	121.7	C17—C22—H22	120.5
N2—C6—C5	131.51 (12)	C24—C23—H23A	109.5
N2—C6—C1	105.68 (11)	C24—C23—H23B	109.5
C5—C6—C1	122.80 (12)	H23A—C23—H23B	109.5
N1—C7—N2	112.80 (11)	C24—C23—H23C	109.5
N1—C7—C8	124.00 (11)	H23A—C23—H23C	109.5
N2—C7—C8	123.12 (11)	H23B—C23—H23C	109.5
O1—C8—C7	109.64 (10)	N8—C24—C23	178.40 (18)
O1—C8—H8A	109.7	C26—C25—H25A	109.5
C7—C8—H8A	109.7	C26—C25—H25B	109.5
O1—C8—H8B	109.7	H25A—C25—H25B	109.5
C7—C8—H8B	109.7	C26—C25—H25C	109.5
H8A—C8—H8B	108.2	H25A—C25—H25C	109.5
O1—C9—C10	106.29 (10)	H25B—C25—H25C	109.5
O1—C9—H9A	110.5	N9—C26—C25	180.000 (3)
			
N1^i^—Co—N1—C7	−63.92 (10)	O1—C9—C10—N4	141.87 (12)
N3—Co—N1—C7	60.12 (11)	C10—N4—C11—C12	178.52 (17)
N3^i^—Co—N1—C7	166.22 (10)	C10—N4—C11—C16	0.22 (15)
N1^i^—Co—N1—C1	116.01 (11)	N4—C11—C12—C13	−177.85 (17)
N3—Co—N1—C1	−119.95 (10)	C16—C11—C12—C13	0.2 (3)
N3^i^—Co—N1—C1	−13.86 (11)	C11—C12—C13—C14	0.3 (3)
N1—Co—N3—C10	−29.43 (11)	C12—C13—C14—C15	−0.3 (3)
N1^i^—Co—N3—C10	98.12 (10)	C13—C14—C15—C16	−0.3 (3)
N3^i^—Co—N3—C10	−141.21 (11)	N4—C11—C16—C15	177.74 (13)
N1—Co—N3—C16	148.33 (10)	C12—C11—C16—C15	−0.8 (2)
N1^i^—Co—N3—C16	−84.11 (11)	N4—C11—C16—N3	−0.97 (15)
N3^i^—Co—N3—C16	36.55 (10)	C12—C11—C16—N3	−179.47 (14)
C7—N1—C1—C2	177.34 (14)	C14—C15—C16—C11	0.8 (2)
Co—N1—C1—C2	−2.6 (2)	C14—C15—C16—N3	179.14 (14)
C7—N1—C1—C6	−0.67 (13)	C10—N3—C16—C11	1.34 (14)
Co—N1—C1—C6	179.40 (8)	Co—N3—C16—C11	−176.71 (9)
C6—C1—C2—C3	0.0 (2)	C10—N3—C16—C15	−177.19 (14)
N1—C1—C2—C3	−177.80 (13)	Co—N3—C16—C15	4.8 (2)
C1—C2—C3—C4	−0.6 (2)	O2—N5—C17—C18	−172.99 (14)
C2—C3—C4—C5	0.3 (2)	O3—N5—C17—C18	6.38 (19)
C3—C4—C5—C6	0.6 (2)	O2—N5—C17—C22	7.5 (2)
C7—N2—C6—C5	−178.36 (14)	O3—N5—C17—C22	−173.11 (13)
C7—N2—C6—C1	0.86 (14)	C22—C17—C18—C19	0.6 (2)
C4—C5—C6—N2	177.88 (13)	N5—C17—C18—C19	−178.90 (12)
C4—C5—C6—C1	−1.2 (2)	C17—C18—C19—C20	−0.1 (2)
C2—C1—C6—N2	−178.38 (12)	C17—C18—C19—N6	177.32 (12)
N1—C1—C6—N2	−0.12 (13)	O5—N6—C19—C18	162.30 (14)
C2—C1—C6—C5	0.92 (19)	O4—N6—C19—C18	−19.0 (2)
N1—C1—C6—C5	179.19 (12)	O5—N6—C19—C20	−20.2 (2)
C1—N1—C7—N2	1.27 (14)	O4—N6—C19—C20	158.52 (15)
Co—N1—C7—N2	−178.79 (8)	C18—C19—C20—O6	173.77 (13)
C1—N1—C7—C8	−175.73 (12)	N6—C19—C20—O6	−3.5 (2)
Co—N1—C7—C8	4.21 (18)	C18—C19—C20—C21	−2.18 (18)
C6—N2—C7—N1	−1.38 (15)	N6—C19—C20—C21	−179.48 (12)
C6—N2—C7—C8	175.65 (12)	O6—C20—C21—C22	−171.77 (14)
C9—O1—C8—C7	−77.34 (13)	C19—C20—C21—C22	4.36 (19)
N1—C7—C8—O1	−26.34 (18)	O6—C20—C21—N7	7.1 (2)
N2—C7—C8—O1	156.97 (11)	C19—C20—C21—N7	−176.76 (12)
C8—O1—C9—C10	153.52 (10)	O7—N7—C21—C22	−140.26 (15)
C16—N3—C10—N4	−1.25 (14)	O8—N7—C21—C22	37.34 (19)
Co—N3—C10—N4	177.02 (8)	O7—N7—C21—C20	40.78 (19)
C16—N3—C10—C9	179.16 (11)	O8—N7—C21—C20	−141.62 (13)
Co—N3—C10—C9	−2.57 (16)	C20—C21—C22—C17	−4.2 (2)
C11—N4—C10—N3	0.67 (15)	N7—C21—C22—C17	176.93 (12)
C11—N4—C10—C9	−179.76 (12)	C18—C17—C22—C21	1.5 (2)
O1—C9—C10—N3	−38.59 (16)	N5—C17—C22—C21	−179.07 (12)

Symmetry codes: (i) −*x*+1, *y*, −*z*+3/2.

### Hydrogen-bond geometry (Å, °)

**Table:**

*D*—H···*A*	*D*—H	H···*A*	*D*···*A*	*D*—H···*A*
N2—H2N···O6^ii^	0.86 (1)	1.89 (1)	2.6746 (14)	151 (2)
N2—H2N···O5^ii^	0.86 (1)	2.38 (2)	3.0166 (15)	131 (2)
N4—H4N···N8	0.86 (1)	2.05 (1)	2.9040 (18)	172 (2)

Symmetry codes: (ii) *x*, *y*−1, *z*.
